# Contraction Heuristics for Tensor Decision Diagrams

**DOI:** 10.3390/e26121058

**Published:** 2024-12-05

**Authors:** Christian Bøgh Larsen, Simon Brun Olsen, Kim Guldstrand Larsen, Christian Schilling

**Affiliations:** Department of Computer Science, Aalborg University, 9220 Aalborg, Denmarkkgl@cs.aau.dk (K.G.L.)

**Keywords:** tensor network, contraction planning, tensor decision diagram, heuristics, quantum circuit, equivalence checking

## Abstract

In this paper, we study the equivalence problem for quantum circuits: Given two quantum circuits, are they equivalent? We reduce this problem to the contraction problem of a tensor network. The order in which the contraction operations between tensors are applied has a crucial impact on efficiency, which is why many heuristics have been proposed. In this work, we use an efficient representation of tensors as a tensor decision diagram. Since existing contraction heuristics do not perform well in combination with these diagrams, we propose two new contraction heuristics. We demonstrate experimentally that our heuristics outperform other state-of-the-art heuristics. We also demonstrate that our framework yields state-of-the-art performance for equivalence checking.

## 1. Introduction

Interest in quantum computing has rapidly increased in both the scientific community and industry. Quantum computers promise to speed up certain computations to a potentially exponential degree [1]. While quantum computers are currently impractical for industrial use, the rate of improvement is similar to that of early classical computers, and it is expected that in the near future, practically usable quantum computers will exist. In order to enable effective use of quantum computers once they mature, the required tools for supporting quantum computation need to be developed now, e.g., verification of quantum algorithms against specifications [2,3] or efficient simulation [4,5].

A central task in quantum computing is the design and optimization of quantum algorithms, with the prevailing paradigm being quantum circuits. The circuit design typically takes place at a high abstraction level, where arbitrary quantum gates can be used. Since a real quantum computer only has a limited set of quantum gates available, the circuit must then be transformed, or *compiled*, into an equivalent circuit meeting these constraints [6]. In addition, the size of a circuit correlates with the noise that occurs during computation. Since noise is the main challenge to the practical use of quantum computers today, additional optimization steps are applied to reduce the size of the circuit [7]. Quantum compilers thus perform an important and nontrivial task, and it is critical that the circuit they produce is functionally equivalent to the original design [8].

Functional equivalence of two quantum circuits means that, for any input state, the two circuits agree on the resulting output state. There are strong reasons to believe that checking for equivalence is hard: deciding approximate equivalence is QMA-complete [9,10], and deciding exact equivalence is NQP-complete [11]; problems in these complexity classes are widely believed to require exponential computations in the worst case.

In this work, we study the problem of checking the equivalence of quantum circuits. Our work is inspired by recent developments in the field, which we briefly summarize next.

### 1.1. Related Work

**Tensor networks.** A *tensor* is a higher-dimensional generalization of a matrix. A tensor network is a graph with tensors as nodes. As such, a quantum circuit can be naturally modeled as a tensor network where every node is a matrix representing a quantum gate [12].

The most important operation in tensor networks is the contraction of two tensors into one. Iteratively contracting tensors in the network eventually yields a single tensor. Since the order in which the contractions are applied has a large impact on the intermediate size, many heuristics have been proposed to find a good contraction order [13,14], several of which are implemented in the contraction tool cotengra  [15].

**Decision diagrams.** A quantum gate matrix can be represented more succinctly in a symbolic data structure called a *decision diagram*. Several decision diagrams have been proposed in the literature [16,17,18,19,20], and we refer to them uniformly as quantum decision diagrams (QDDs). QDDs may offer exponential compression, but they are no silver bullet. Since QDDs exploit symmetries in the matrix, different matrices/circuits show different compression potential. Analogously, a *tensor decision diagram* (TDD) symbolically represents a tensor [21], which thus generalizes the QDD. TDDs have recently been demonstrated to speed up quantum simulations [4].

**Quantum circuit equivalence.** Checking the equivalence of two quantum circuits $C_{1}$ and $C_{2}$ is a conceptually simple comparison of their characteristic matrices. However, the size of these matrices is exponential in terms of the number of qubits, and thus, this comparison cannot be performed for reasonably sized circuits. Alternatively, the problem reduces to comparing the composition of $C_{1}$ and the adjoint of $C_{2}$ to the identity [9]. By representing gates as QDDs, this “adjoint scheme” can lead to an efficient equivalence check [22]. By performing the involved matrix multiplications in a clever order, this scheme performs even better [23], and it is implemented in the tool qcec  [24]. Efforts to identify a good order using contraction heuristics from tensor networks were not successful [25], at least when restricted to QDDs; in this work, we instead use TDDs for representing tensor networks efficiently, and we shall see that this yields a method that is competitive with the QDD-based approach. Equivalence of *dynamic* quantum circuits was addressed by directly encoding the two circuits as TDDs individually and then checking whether these TDDs are identical, i.e., not using the adjoint scheme that we use [26].

Orthogonal approaches to equivalence checking include ZX-calculus [27], which can sometimes yield results quickly [28]. Alternatively, if the quantum circuits belong to a restricted subclass called the Clifford group, a polynomial-time approach exists [29]. TDDs have been applied to approximate equivalence checking based on computing traces [30].

### 1.2. Contributions

In this paper, we integrate several of the above techniques into a new approach to the equivalence checking of quantum circuits. We represent the quantum circuits as a tensor network with the adjoint scheme described above. When operating on tensor networks, further benefits arise because tensors of different sizes can be contracted, which is not possible with QDDs. To efficiently represent the tensors in the network, we use TDDs.

However, as mentioned above, decision diagrams are not equally efficient on different circuits. Hence, contraction orders that may be beneficial for tensor networks with ordinary tensors may be suboptimal, or even detrimental, to TDD networks (i.e., tensor networks where the tensors are represented as TDDs). Thus, we are in need of new contraction heuristics for TDD networks, and we propose two such heuristics in this work. In our experimental evaluation, we demonstrate that our heuristics outperform other heuristics implemented in cotengra and that our TDD-based approach for equivalence checking is competitive with qcec.

### 1.3. Outline

The rest of this paper is structured as follows: In Section 2, we summarize background information on quantum computing, tensor networks, decision diagrams, and contraction heuristics. In Section 3, we describe our contraction heuristics. In Section 4, we evaluate our contraction heuristics and compare them to state-of-the-art methods. In Section 5, we conclude the paper and discuss future directions.

## 2. Background

In this section, we recall some basic concepts needed to understand the rest of the paper. We start with a short introduction to quantum circuits. Then, we explain tensor networks and how they relate to quantum circuits, followed by describing tensor contraction. After that, we give a brief overview of tensor decision diagrams. Finally, we discuss how to check the equivalence of two quantum circuits.

### 2.1. Quantum Circuits

We briefly recall the main concepts of quantum computing and the quantum circuit model. For a broader introduction, we refer to the literature [31].

The basic unit in quantum computing is the *qubit*, like the bit is to digital computing. While a bit can be in one of two states, 0 or 1, a qubit can be in the basis states |0〉 or |1〉 (using the Dirac/BraKet notation), or it can be in a linear combination of these; in the latter case, the qubit is said to be in a superposition. Formally, a qubit $|q_{0}\rangle$ is described as $|q_{0}\rangle =\alpha |0\rangle +\beta |1\rangle .$ The values α,β∈C are the amplitudes, and they must satisfy the side condition ${\left|\alpha \right|}^{2}+{\left|\beta \right|}^{2}=1$. Measuring $|q_{0}\rangle$ results in the basis state |0〉 with probability ${\left|\alpha \right|}^{2}$ and in the basis state |1〉 with probability ${\left|\beta \right|}^{2}$.

We generalize the notion of a *quantum state* to multiple qubits. Since a quantum state may be in a superposition of each combination of basis states, it has $2^{n}$ amplitudes for *n* qubits. A quantum state can also be represented as a state vector of the amplitudes (e.g., ${\left[\alpha ,\beta \right]}^{T}$ for $|q_{0}\rangle$ above).

**Example** **1** (Quantum state)**.** *Consider a quantum state of 3 qubits, $q_{0}$, $q_{1}$, and $q_{2}$. A state vector for such a state is*

$${\left[\alpha_{000},\alpha_{001},\ldots ,\alpha_{111}\right]}^{T}\;s.t.\sum_{i}{\left|\alpha_{i}\right|}^{2}=1,$$

*which represents a linear combination of all eight basis states:*

$$|q_{0}q_{1}q_{2}\rangle =\alpha_{000}|000\rangle +\alpha_{001}|001\rangle +\cdots +\alpha_{111}|111\rangle$$

*The probability of measuring the basis state |i〉 is $|\alpha_{i}{|}^{2}$.*

Quantum computation aims at transforming quantum states. A common framework to express these transformations is the quantum circuit model. At the lowest level, a *quantum gate* (henceforth gate) transforms a quantum state into a new quantum state. It is generally sufficient to restrict oneself to gates involving at most two qubits. Each gate corresponds to the application of a linear map with an associated complex unitary matrix.

**Example** **2** (Quantum gate)**.** *Given a quantum state over a single qubit $q_{0}$, the Hadamard gate H has the associated matrix (we abuse notation and write H both for the gate and the associated matrix).*

$$H=\frac{1}{\sqrt{2}}\left[\begin{array}{cc} 1 & 1\\ 1 & -1 \end{array}\right].$$

*The Hadamard gate transforms the basis state |0〉=1·|0〉+0·|1〉 into a superposition:*

$$H|0\rangle =\frac{1}{\sqrt{2}}\left[\begin{array}{cc} 1 & 1\\ 1 & -1 \end{array}\right]\cdot \left[\begin{array}{c} 1\\ 0 \end{array}\right]=\frac{1}{\sqrt{2}}\left[\begin{array}{c} 1\\ 1 \end{array}\right]=\frac{1}{\sqrt{2}}|0\rangle +\frac{1}{\sqrt{2}}|1\rangle$$

**Figure 1:**
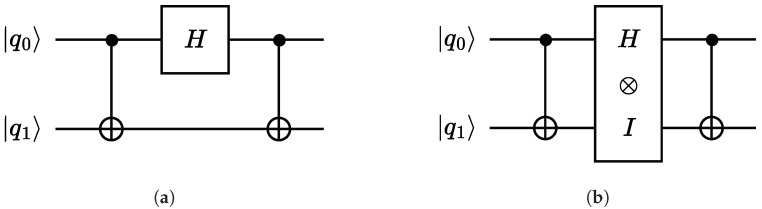
A simple quantum circuit. (**a**) A two-qubit quantum circuit consisting of two CNOT-gates and one Hadamard gate; (**b**) the same circuit after expanding the Hadamard gate *H* using the tensor product.

Similar to a classical digital circuit, we can combine quantum gates into a *quantum circuit* (henceforth circuit). Figure 1a shows an example circuit involving three gates on two qubits. In the example, the CNOT gate acts on two qubits, while the *H* gate acts on only one qubit. To compute the effect of the CNOT gate on a two-qubit vector (and hence a four-dimensional state vector) $|q_{0}\rangle$, we can simply multiply with the associated 4×4-matrix CX as before: $CX\cdot |q_{0}\rangle$. However, since the *H* gate only acts on one qubit, we cannot apply matrix multiplication directly. As an intermediate step, we insert an artificial identity matrix on the lower qubit wire, as shown in Figure 1b.

In general, we need to compute the effect of multiple parallel gates with qubit counts $n_{1},\ldots ,n_{k}$, respectively, to a state vector involving *n* qubits, where $n_{1}+\cdots +n_{k}=n$. This is achieved by taking the tensor product of the gate matrices.

**Example** **3** (Quantum circuit)**.** *The effect of applying a Hadamard gate to the first qubit of a two-qubit system (as in Figure 1) can be computed as the tensor product*

$$H⊗I=\frac{1}{\sqrt{2}}\left[\begin{array}{cc} 1 & 1\\ 1 & -1 \end{array}\right]⊗\left[\begin{array}{cc} 1 & 0\\ 0 & 1 \end{array}\right]=\frac{1}{\sqrt{2}}\left[\begin{array}{cccc} 1 & 0 & 1 & 0\\ 0 & 1 & 0 & 1\\ 1 & 0 & -1 & 0\\ 0 & 1 & 0 & -1 \end{array}\right].$$

*Thus, the whole effect of the circuit in Figure 1a is CX·(H⊗I)·CX, or*

$$\left[\begin{array}{cccc} 1 & 0 & 0 & 0\\ 0 & 1 & 0 & 0\\ 0 & 0 & 0 & 1\\ 0 & 0 & 1 & 0 \end{array}\right]\cdot \frac{1}{\sqrt{2}}\left[\begin{array}{cccc} 1 & 0 & 1 & 0\\ 0 & 1 & 0 & 1\\ 1 & 0 & -1 & 0\\ 0 & 1 & 0 & -1 \end{array}\right]\cdot \left[\begin{array}{cccc} 1 & 0 & 0 & 0\\ 0 & 1 & 0 & 0\\ 0 & 0 & 0 & 1\\ 0 & 0 & 1 & 0 \end{array}\right]=\frac{1}{\sqrt{2}}\left[\begin{array}{cccc} 1 & 0 & 0 & 1\\ 0 & 1 & 1 & 0\\ 0 & 1 & -1 & 0\\ 1 & 0 & 0 & -1 \end{array}\right].$$

### 2.2. Tensor Networks

Matrices are sufficient to express quantum gates (and circuits). A *tensor* is a generalization to higher dimensions. We can thus use tensors to represent both quantum gates (two-dimensional matrices) and quantum states (one-dimensional vectors).

**Figure 2:**
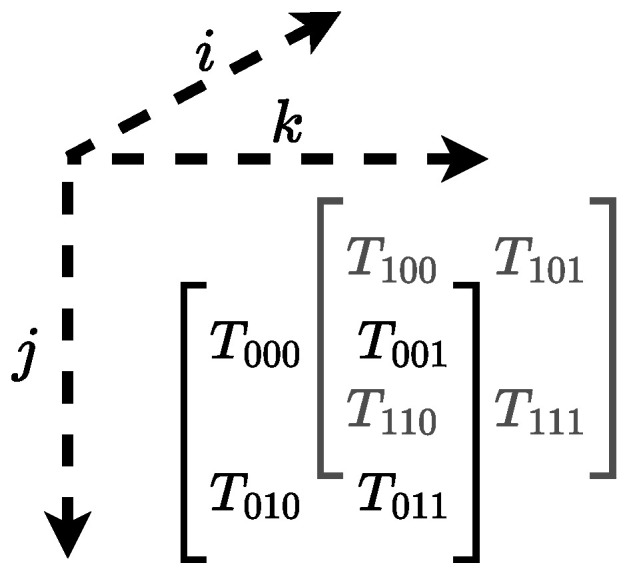
Example of a three-dimensional tensor with corresponding indices *i*, *j*, and *k*.

Tensors have an associated index vector I, where each entry is an *index variable* for the corresponding dimension. In our context, a tensor will have an index for each qubit, and the corresponding index variable has two possible values, one for each of the two basis states (|0〉 and |1〉). Figure 2 shows a three-dimensional tensor together with its indices.

The elements in our tensors are complex numbers, so tensors have the form $T\in C^{2^{\left|I\right|}}$. We will use lowercase symbols to denote a concrete index vector, e.g., $\vec{i}\in {\left\{0,1\right\}}^{\left|I\right|}$.

**Figure 3:**
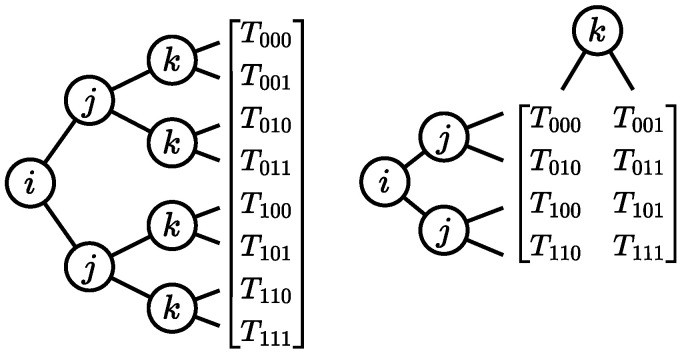
A tensor with three index variables *i*, *j*, and *k*, equivalently depicted both as a column vector and as a matrix, showing how indices correspond to the position of the elements.

**Example** **4** (Tensor)**.** *Let $I={\left[i,j,k\right]}^{T}$ be a three-dimensional vector of index variables. Consider the tensor T depicted in Figure 3, which is shown in two alternative (but equivalent) representations. A (concrete) index vector is $\vec{i}={\left[0,0,0\right]}^{T}$, with the corresponding tensor entry $T_{\vec{i}}=T_{000}$.*

As shown in Figure 3, the placement of elements is determined by the indices, and the alignment with rows and columns does not have any meaning for the tensor.

Just as gates can be combined into quantum circuits, we can combine multiple tensors into *tensor networks*. A tensor network is a graph whose vertices are tensors and whose edges connect tensors if they share an index. The same index can only be shared by two tensors, and we hence use the words *edges* and *indices* interchangeably in this context.

Circuits contain input and output wires which are only connected to one gate, the other end being free (unless a state vector is supplied as an input). Similarly, our tensor networks also have dedicated input and output indices which are only connected to one tensor; we refer to these as outer edges. Formally:

**Definition** **1** (Tensor network)**.** *A tensor network is an undirected graph $G=(V,E,E_{\mathrm{outer}},S)$, where $V\subseteq {⋃}_{m\in N}C^{2^{m}}$ is the finite set of vertices, each of which is a tensor of some index dimension m; S is the finite set of index variables; $E\subseteq V\times V\times 2^{S}$ is a set of (inner) edges; and $E_{\mathrm{outer}}\subseteq V\times 2^{S}$ is a set of outer edges. Each edge is labeled with a nonempty set of index variables.*

**Figure 4:**
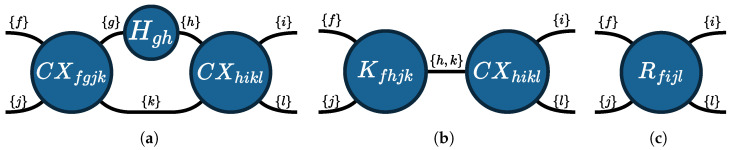
The tensor network of the circuit in Figure 1a being contracted into a single tensor in two contraction steps. The edges between two tensors are labeled with the shared indices. (**a**) Original tensor network; (**b**) contraction of $CX_{fgjk}$ and $H_{gh}$; (**c**) final result.

**Example** **5** (Tensor network)**.** *Consider the tensor network $\left(V,E,E_{\mathrm{outer}},S\right)$ depicted in Figure 4a. It consists of three tensors: $V=\{CX_{fgjk},H_{gh},CX_{hikl}\}$. The tensors are the matrices corresponding to the respective gates, as indicated by the names. (For convenience, we use the naming convention that a tensor is named after its gate with its indices in the subscript.) The index set S of the tensor network is determined by the possible index variables of all tensors in the tensor network, here S={f,g,h,i,j,k,l}. The outer edges $E_{\mathrm{outer}}$ are the edges that only attach to one tensor, i.e., those labeled with f, i, j, and l. The remaining edges each connect two of the tensors. For our tensor network, we have the following sets of edges:*

$$\begin{array}{cc} E & =\{\left(CX_{fgjk},H_{gh},\left\{g\right\}\right),\left(CX_{fgjk},CX_{hikl},\left\{k\right\}\right),\left(H_{gh},CX_{hikl},\left\{h\right\}\right)\}\\ E_{\mathrm{outer}} & =\{\left(CX_{fgjk},\left\{f\right\}\right),\left(CX_{fgjk},\left\{j\right\}\right),\left(CX_{hikl},\left\{i\right\}\right),\left(CX_{hikl},\left\{l\right\}\right)\} \end{array}$$

Similarly to how the gate matrices can be combined into a single matrix representation of a circuit by matrix multiplication, the tensors of the tensor network can be combined into a single tensor representing the same circuit. The operation for combining tensors is *tensor contraction*, which generalizes matrix multiplication. The operation multiplies the elements of each tensor with corresponding index values together, summing over shared indices.

**Definition** **2** (Tensor contraction)**.** *Let $T_{\vec{I},\vec{J}}$ and $T_{\vec{J},\vec{K}}^{\prime }$ be two tensors with their index variables split into shared variables $\vec{J}$ and unique variables $\vec{I}$ and $\vec{K}$, respectively. The result of the* (tensor) contraction *is the tensor*

$$R_{\vec{i},\vec{j}}=\sum_{\vec{x}\in {\left\{0,1\right\}}^{|\vec{J}|}}T_{\vec{i},\vec{x}}\cdot T_{\vec{x},\vec{j}}^{\prime }.$$

**Example** **6** (Tensor contraction)**.** *Recall the tensor network from Figure 4a. By contracting the tensors $CX_{fgjk}$ and $H_{gh}$ over the {g} edge, we obtain the tensor (depicted in Figure 4b)*

$$K_{fhjk}=\sum_{x\in \{0,1\}}CX_{fxjk}\cdot H_{xh}.$$

*Next, by contracting the tensors $K_{fhjk}$ and $CX_{hikl}$ over the {h,k} edge, we obtain the tensor (depicted in Figure 4c)*

$$R_{fijl}=\sum_{x,y\in \{0,1\}}K_{fxjy}\cdot CX_{xiyl}.$$

### 2.3. Tensor Decision Diagrams

A tensor decision diagram (TDD) is a symbolic representation of a tensor. TDDs exploit symmetries to avoid storing (multiples of) the same tensor entries several times.

**Definition** **3** (Tensor decision diagram [21])**.** *A* tensor decision diagram *is a weighted, rooted, and directed acyclic graph $\left(V,E,\mathrm{idx},w_{g}\right)$ over a set of indices S, defined as follows:*

- *$V=V_{N}\cup \left\{v_{T}\right\}$ is the set of nodes, where $V_{N}$ is a finite set of non-terminal nodes, and $v_{T}$ is the terminal node.*
- *$E\subseteq V_{N}\times V\times C\times \left\{\mathrm{low},\mathrm{high}\right\}$ is the set of weighted edges. Each node in $V_{N}$ has exactly one outgoing low edge and one outgoing high edge.*
- *$\mathrm{idx}:V_{N}\to S$ assigns an index from the index set S to each non-terminal node.*
- *$w_{g}\in C$ is a global weight associated with the root node.*

While there are multiple TDD representations of the same tensor, in practice, one uses a normalization procedure such that for each tensor there exists a corresponding canonical TDD. For further details on TDDs, we refer to the original presentation [21].

**Figure 5:**
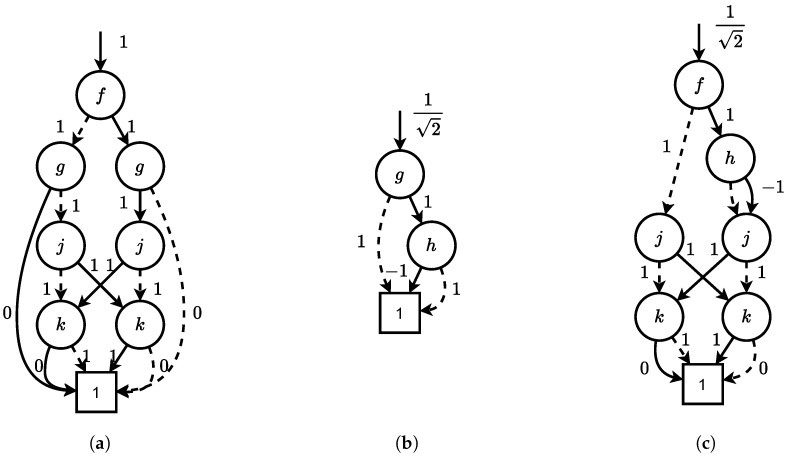
TDDs of two tensors in Figure 4, and the resulting TDD after contraction. (**a**) TDD for $CX_{fgjk}$; (**b**) TDD for $H_{gh}$; (**c**) TDD for $K_{fhjk}$.

**Example** **7.** *Figure 5 shows the TDDs for the tensors from Figure 4 before and after contraction. The representation size after contraction (7 nodes) is smaller than before contraction (8+3 nodes).*

### 2.4. Quantum Circuit Equivalence

We now turn to the equivalence problem for quantum circuits. First, we consider again the quantum circuit model, and later generalize the idea to tensor networks.

Recall that each circuit corresponds to the application of a single unitary matrix. In this light, we define the equivalence of two quantum circuits via their corresponding matrices.

**Definition** **4** (Quantum circuit equivalence)**.** *Two circuits $C_{1}$ with $n_{1}$ qubits and $C_{2}$ with $n_{2}$ qubits and with matrix representations $U_{C_{1}}$ and $U_{C_{2}}$, respectively, are* functionally equivalent*, written $C_{1}\equiv C_{2}$, if and only if $n_{1}=n_{2}$, and we have that*

$$\exists \theta \in \left[0,2\pi \right):U_{C_{1}}=e^{i\theta }\cdot U_{C_{2}}.\;$$

Note that the matrices only need to be identical up to a factor $e^{i\theta }$ called the global phase; this is due to the quantum phenomenon that two states can only be experimentally distinguished up to this factor.

However, computing the matrices of two *n*-qubit circuits is expensive, since the matrices have $2^{n}\times 2^{n}$ (i.e., exponentially many) entries. We thus turn to potentially cheaper ways of determining equivalence.

Since unitary matrices are invertible, all gates (and hence circuits) are reversible. We denote the adjoint of a matrix *U* by $U^{†}$. Then, we get the following alternative [22]:

**Proposition** **1.** *Given two n-qubit circuits $C_{1}$ and $C_{2}$, we have:*

$$C_{1}\equiv C_{2}\Leftrightarrow \exists \theta \in \left[0,2\pi \right):U_{C_{1}}\cdot U_{C_{2}}^{†}=e^{i\theta }\cdot I.\;$$

In other words, we can multiply the first circuit with the adjoint of the second circuit and compare the result to the identity matrix. (To simplify the presentation, we ignore the global phase in the following and only compare to the identity.)

While there is no immediate advantage in using this combined circuit setup as opposed to checking whether the matrix representations of two circuit are equivalent, there is a possibility of exploiting the fact that gates from one circuit are negated by gates from the inverse of the other circuit. In particular, if $C_{1}$ and $C_{2}$ are identical, then the last gate of $C_{1}$ and the first gate of $C_{2}$ cancel out, and this holds true for all other pairs of gates.

Additionally, there exist alternative data structures (quantum decision diagrams (QDDs)) to represent quantum matrices, potentially avoiding the exponential space requirements. Hence, by performing matrix multiplication on gates from both circuits, we may be able to maintain such a data structure without an exponential blow-up and thus obtain an efficient procedure for checking the equivalence of quantum circuits. It should be noted that there is no guarantee that this blow-up can generally be avoided. However, it has been demonstrated that it can be avoided in many practical cases [23].

For tensors and tensor networks, we can apply a similar approach. Initially, we transform the two circuits $C_{1}$ and $C_{2}$ into two tensor networks. This is a standard construction (e.g., [32]) using the matrix as the tensor for each gate. Next, the straightforward approach would be to contract both tensor networks and finally compare the two resulting tensors for equality (up to a global phase). Again, we can apply the same trick as in Proposition 1 and build a single tensor network with $C_{2}$ inverted. Then, we contract that tensor network and in the end check whether the resulting tensor is the identity (up to a global phase).

As with quantum circuits, we do not necessarily gain anything from this construction if we use an explicit tensor representation. However, just as with QDDs, when representing tensors with TDDs, we can often avoid an exponential blow-up in practice.

Note that working with tensors as compared to quantum gates has potential benefits. Gates are represented as matrices, and thus, contraction corresponds to matrix multiplication, for which the two matrices need to have the same dimension. General tensor contraction can be performed on any subset of shared indices. This allows one to partially contract tensors resulting from parallel gates in a quantum circuit, whereas a gate-based approach has to multiply with all of the gates at the same time. For example, in Figure 4, we contracted a CX tensor with an *H* tensor, whereas a gate-based approach would have to insert an identity gate (using a tensor product) as in Figure 1b.

### 2.5. Contraction Heuristics for Tensor Networks and TDD Networks

Recall that, to contract a tensor network to a single tensor, we just have to repeatedly apply the contraction operation for two tensors with shared indices. The sequence in which tensors are contracted is called the *contraction plan*. Each contraction step will reduce the number of tensors in the network by one, thus eventually resulting in one tensor representing the whole network. This final tensor is unique, independent of the contraction plan, but the intermediate tensors are not. Thus, the contraction plan has a large impact on the size of the intermediate representation. Correspondingly, a significant amount of work has gone into finding good heuristics to choose a good contraction plan.

**Example** **8** (Tensor contraction (alternative))**.** *Recall the tensor network from Figure 4a. In Example 6, we chose one of three possible contraction plans. The two alternatives are*

*1.*  *Contract $CX_{hikl}$ and $H_{gh}$ via edge h to obtain $K_{gikl}$. Then contract $CX_{fgjk}$ and $K_{gikl}$ via edges {g,k}.*
*2.*  *Contract $CX_{fgjk}$ and $CX_{hikl}$ via edge k to obtain $K_{fgjhil}$. Then contract $H_{gh}$ and $K_{fghjil}$ via edges {g,h}.*

Existing heuristics have been designed under the assumption that the tensors are explicitly stored as high-dimensional vectors. In this work, we instead represent tensors implicitly as TDDs. TDDs representing tensors with the same number of elements may have vastly different representation sizes. Hence, it is not clear whether the existing heuristics will perform equally well in our scenario. The gap in representation size can be exponential; for example, the TDD representing the identity tensor is linear in the number of qubits, whereas the explicit tensor has exponentially many entries. The main objective of this paper is to find a good contraction plan when the tensors are represented as TDDs; in particular, we aim for small intermediate TDD representations.

**Figure 6:**
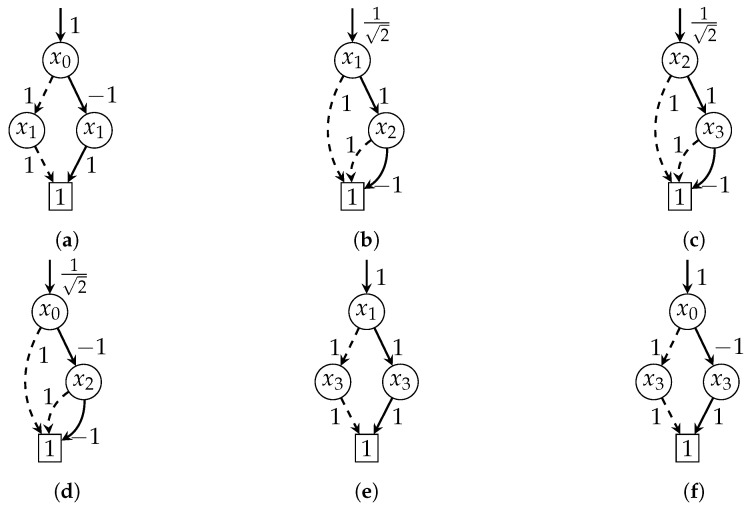
TDDs for the tensor network $\overset{x_{0}}{-}Z\overset{x_{1}}{-}H_{1}\overset{x_{2}}{-}H_{2}\overset{x_{3}}{-}$. (**a**) TDD for $\overset{x_{0}}{-}Z\overset{x_{1}}{-}$; (**b**) TDD for $\overset{x_{1}}{-}H_{1}\overset{x_{2}}{-}$; (**c**) TDD for $\overset{x_{2}}{-}H_{2}\overset{x_{3}}{-}$; (**d**) the resulting TDD when contracting $\overset{x_{0}}{-}Z\overset{x_{1}}{-}H_{1}\overset{x_{2}}{-}$ via $x_{1}$; (**e**) the resulting TDD when contracting $\overset{x_{1}}{-}H_{1}\overset{x_{2}}{-}H_{2}\overset{x_{3}}{-}$ via $x_{2}$; (**f**) the (unique) resulting TDD when contracting the whole tensor network.

**Example** **9.** *To reduce the complexity, we now simplify our running example to use only unary gates. Figure 6 shows the TDDs for the tensor network consisting of a Z gate followed by two H gates. Clearly, the two H gates cancel each other out, and thus, the circuit is equivalent to a Z gate.*

*Initially, we can choose between two contractions: (1) of the Z and the first H gate or (2) of the first and the second H gate. The results are shown in Figure 6d and Figure 6e, respectively. As can be seen, the first contraction has a smaller intermediate representation. Thus, this contraction would be preferred for us. The final TDD is equivalent to the TDD of the Z gate, as expected.*

In the simplest case, a (offline) contraction heuristic works in two phases. In the *planning phase*, a contraction plan is found. In the *execution phase*, the plan is executed. In this paper, we focus on (online) heuristics which alternately perform one planning step (i.e., identify the next contraction step) and one execution step (i.e., execute the contraction step). The advantage of such an online heuristic is that it does not have to predict what happens after the next step.

## 3. Contraction Heuristics for TDD Networks

In this section, we present two contraction heuristics for TDD networks. Both heuristics work *online*, i.e., they interleave planning and execution by only determining the next contraction step and executing it immediately.

The first heuristic, which we call the *lookahead method*, is a greedy method which results in a good contraction order but requires additional computations in the planning steps. Motivated by this success, the second heuristic, which we call the *counting method*, approximates the first heuristic; as a result, it still produces an overall good contraction order but also uses fast planning steps.

### 3.1. Lookahead Method

Our first heuristic method follows a greedy policy. In the planning step, each possible next contraction of two connected TDDs is evaluated, and the contraction with the smallest resulting TDD is selected for the next execution step. Consequently, we call this heuristic the *lookahead method*.

The planning steps of the lookahead method seem computationally expensive. In particular, there are concerns regarding the cost of the (hypothetical) contractions in a single planning step and that these computations have to be repeated in each planning step. Fortunately, there are a number of reasons why the method still works favorably in practice. Below we argue that the number of contractions is limited, most of the contractions take place in the first planning step, where computations are cheap, and only very few updates are required in each of the later planning steps.

Let *m* be the number of TDDs in the initial TDD network. First, while there are $O(m^{2})$ pairs of TDDs to contract in the worst case, tensor networks obtained from quantum circuits, which typically only contain unary and binary gates, are sparsely connected. Hence, the number of connected pairs is ≤4m, and the quadratic worst case is not observed in our scenario. Second, only the very first planning step requires many contractions. After any execution step of contracting two TDDs $D_{1}$ and $D_{2}$, all other hypothetical contraction results from the previous planning step that involve neither $D_{1}$ nor $D_{2}$ remain valid. Hence, by storing the previous contraction results, planning steps in later iterations involve only a few new computations. Third, contractions in the first planning step are often efficient, since they correspond to the combination of two quantum gates. Even writing out the matrix representation (a 4×4 matrix in the worst case) is cheap.

We summarize the lookahead method in Algorithm 1. The algorithm maintains a priority queue (*Q*), which stores pairs (k,v) of keys *k* (natural numbers) and values *v*. The values are pairs of TDDs, while the keys are the size of the TDD resulting from their contraction. Note that the queue can store multiple values for the same key. Moreover, note that a priority queue allows for efficient enqueue and remove operations (O(logℓ) with *ℓ* elements in the queue), and an O(1)remove_minimum operation. First, we initialize *Q* with each possible pair of TDDs for contraction (which can be easily obtained from the layout of the quantum circuit), where we store the resulting size if we were to contract these TDDs.

In each iteration of the contraction loop (line 6), we start with a planning step that selects two TDDs $D_{1}$ and $D_{2}$ whose contraction result is minimal. In case of multiple options (line 7), any tie breaker can be used (we use the implementation of *Q*). In the execution step, we execute the contraction to obtain the resulting TDD $D_{3}$. Finally, we need to update *Q* because TDDs $D_{1}$ and $D_{2}$ do not exist anymore, and instead, we establish connections with the new TDD $D_{3}$. Note that the loop in line 9 iterates over the whole queue, which takes O(ℓ) steps (where *ℓ* is the number of elements in the queue).
**Algorithm 1** Lookahead method
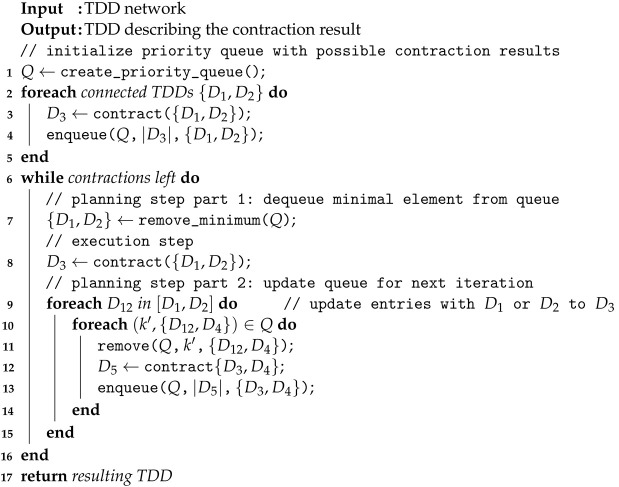


In our implementation, instead of only storing the size and then recomputing the contraction $D_{3}$ from $\left\{D_{1},D_{2}\right\}$, we also directly store the TDD $D_{3}$ in the queue. This way, we avoid recomputing the contraction later. While this seems expensive in terms of memory consumption, the overall memory consumption is typically dominated by the most expensive (single) contraction results, which would already fail in the lookahead step before even getting to the contraction step.

We conclude the algorithm description with an example:

**Example** **10.** *We run Algorithm 1 on the TDD network from Example 9 (Figure 6). Initially, the priority queue contains the following key-value pairs: $Q_{0}=\left[3\mapsto \left\{\left\{Z,H_{1}\right\}\right\},\;4\mapsto \left\{\left\{H_{1},H_{2}\right\}\right\}\right]$. In this case, the lookahead method will execute the contraction of Z and $H_{1}$ (as it results in the smaller TDD) to the TDD $ZH_{1}$. Since $H_{1}$ is part of both pairs in the queue, both pairs will be removed from the queue, and only the final contraction will be inserted: $Q_{1}=\left[4\mapsto \left\{\left\{ZH_{1},H_{2}\right\}\right\}\right]$. Finally, this contraction is executed and the algorithm terminates.*

### 3.2. Counting Method

While we argued that the lookahead method is not as expensive as it seems at first glance, the planning time is still significant and dominates the total contraction time, specifically for circuits with many gates. In particular, the most expensive contractions still have to be evaluated but are then not actually applied.

When we evaluated the lookahead method, we observed a pattern that the next contraction would often happen between two TDDs that have not been contracted in a long time. Our intuition is that, in many cases, the resulting TDD is bigger than both input TDDs, and thus, one should often favor small TDDs.

Thus, to imitate a typical contraction order as obtained from the lookahead method, our second heuristic method is designed to avoid the redundant tentative contractions and simply rank TDDs based on counting how long they have not been involved in any contraction. Accordingly, we call our second heuristic method the *counting method*.

We summarize the lookahead method in Algorithm 2. Instead of a priority queue, the algorithm only requires a standard first-in-first-out (FIFO) queue (*Q*), for which enqueue and dequeue are O(1) operations. The order in which to initialize the queue is arbitrary (in our implementation, we select the order we obtain from iterating over the network).
**Algorithm 2** Counting method
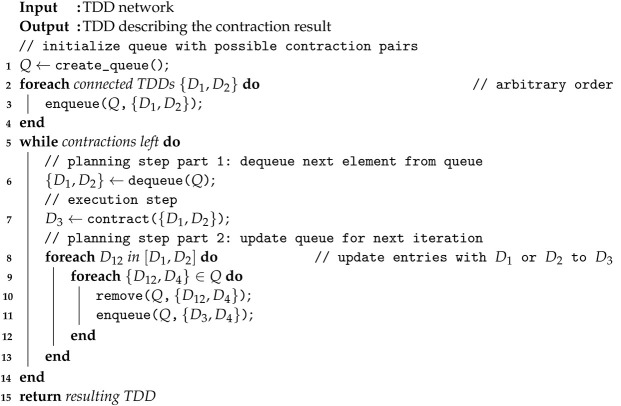


The first planning step simply dequeues the pair $\left\{D_{1},D_{2}\right\}$ from *Q* that has been in the queue for the longest time. The execution step computes the contraction, resulting in TDD $D_{3}$. Finally, we need to move all other pairs involving the TDDs $D_{1}$ and $D_{2}$ to the end of the queue and update these entries to the new TDD $D_{3}$. We note that the corresponding loop in line 8 can be implemented efficiently (i.e., without iterating over the whole queue) with little overhead by implementing the queue as a doubly linked list and storing for each TDD *D* all pointers to pairs involving *D*.

We again conclude the algorithm description with the same example as before:

**Example** **11.** *As in Example 10, we run Algorithm 2 on the TDD network from Example 9 (Figure 6). Initially, the queue Q contains the following TDD pairs (note that the order is arbitrary): $Q_{0}=\left[\left\{Z,H_{1}\right\},\;\left\{H_{1},H_{2}\right\}\right]$. In this case, the counting method will execute the contraction of Z and $H_{1}$ (as it comes first in the queue) to the TDD $ZH_{1}$. Again, since $H_{1}$ is part of both pairs in the queue, both pairs will be removed from the queue, and only the final contraction will be inserted: $Q_{1}=\left[\left\{ZH_{1},H_{2}\right\}\right]$. Finally, this contraction is executed and the algorithm terminates.*

## 4. Evaluation

In this section, we report on the evaluation results of our contraction heuristics when applied to checking the equivalence of quantum circuits. First, we describe our implementation and the benchmark problems we use in the evaluation. Next, we compare our heuristics to other state-of-the-art contraction heuristics implemented in the tool cotengra [15]. Finally, we compare our approach to checking the equivalence of quantum circuits to the tool qcec [24], which has the same aim.

### 4.1. Experimental Setup

Our implementation consisted of three components. First, we implemented a TDD library in C++ for which we substantially extended a preliminary implementation (we extended the preliminary implementation from https://github.com/Veriqc/TDD_C, accessed on 25 May 2024). Second, we implemented our contraction heuristics in C++. Third, we implemented our equivalence checking approach in Python for easy integration with cotengra (see below).

All experiments were run on an Intel i5-14600KF CPU with 27 GB RAM and a GeForce RTX 4070 Super 12 GB GPU. The operating system was Ubuntu 20.04, and we used C++17 and Python 3.9.

To evaluate our approach, we use the application to check equivalence of quantum circuits. For this purpose, we choose quantum circuits from the MQT Bench [33] benchmark suite, which contains many well-known quantum circuits on four different levels of a compilation pipeline. In our evaluation, we select the *algorithmic* (first) level and the *target-dependent* (third) level, which differ significantly in their gate sets and circuit layouts, making the task of equivalence checking challenging.

In particular, we use the following quantum circuits (short explanations are available online (https://www.cda.cit.tum.de/mqtbench/benchmark_description, accessed on 1 November 2024): Deutsch-Jozsa algorithm (DJ), Greenberger-Horne-Zeilinger state preparation (GHZ), graph state preparation (GS), quantum Fourier transformation applied to entangled qubits (QFTE), real amplitudes ansatz with random parameters (RAR), and W state preparation (WS). All circuits have a parametric number of qubits and are available with varying sizes. This allows us to study the scalability in the number of qubits.

### 4.2. Evaluation: Lookahead and Counting Heuristics

In the first experiment, we evaluate our two proposed heuristics. As discussed before, we should expect that the lookahead heuristic spends significantly time on the contraction planning because it tries out all possible next contractions before selecting the best one. The counting heuristic was motivated because it circumvents this cost.

**Figure 7:**
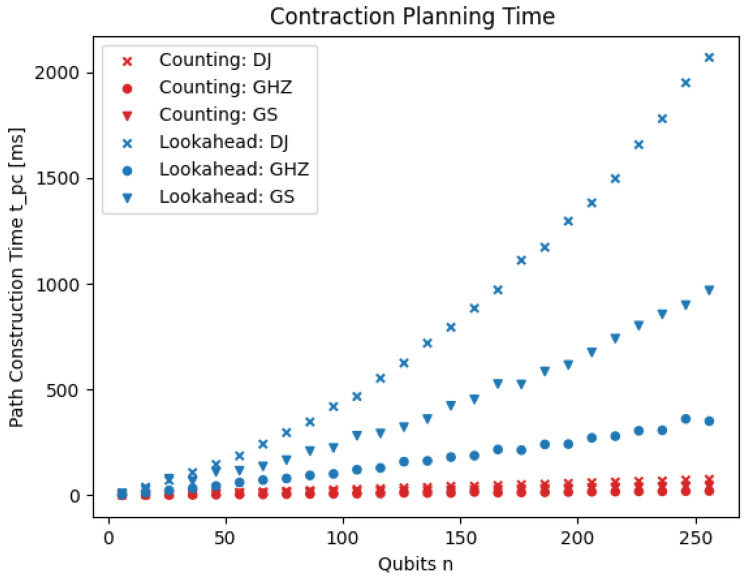
Contraction planning times of the lookahead heuristic and the counting heuristic. The times (y-axis) are shown for three different families of quantum circuits (different markers) and for varying numbers of qubits (x-axis).

Figure 7 shows that this intuition is indeed correct. The planning time of the counting heuristic is significantly shorter compared to the lookahead heuristic.

**Figure 8:**
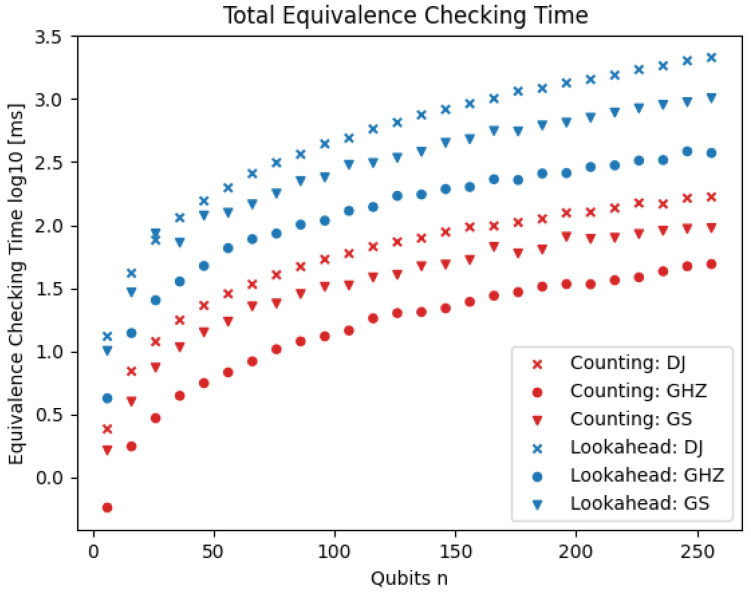
Total equivalence checking time using the lookahead and the counting heuristics. The times (y-axis; log-scale) are shown for three different families of quantum circuits (different markers) and for varying numbers of qubits (x-axis).

Figure 8 shows the total time for equivalence checking with both heuristics. The counting heuristic clearly outperforms the lookahead heuristic (note the log scale). Consequently, we mainly use the counting heuristic in later experiments.

### 4.3. Evaluation: *cotengra* Contraction Heuristics

In this experiment, we compare our counting heuristic to other contraction heuristics from cotengra [15], a state-of-the-art contraction planning tool for tensor networks implemented in Python. Based on prior experimentation, we select the two heuristics that perform best for our setup: *Betweenness* and *RandomGreedy*. The Betweenness heuristic is based on edge betweenness centrality (a graph-theoretic concept) and identifies graph communities in order to perform contractions in these communities first. The RandomGreedy heuristic samples random contraction plans multiple times and selects the best plan according to the sum of the expected amount of floating-point operations of the entire plan.

**Figure 9:**
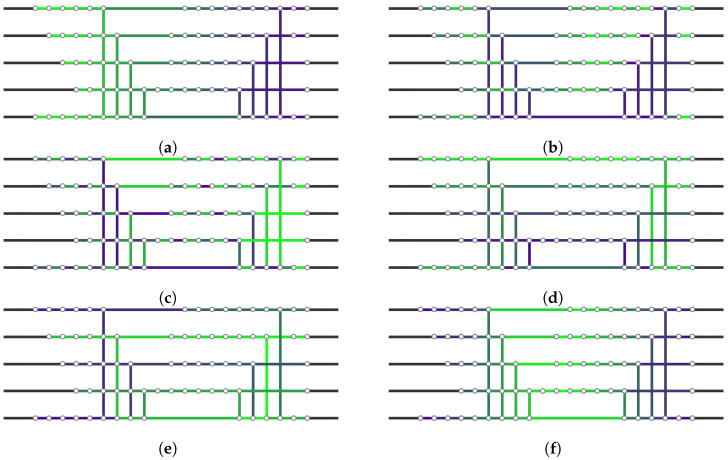
Visualization of different contraction orders on the DJ circuit. The contraction order is encoded in the color spectrum from green (contracted first) to purple (contracted last). (**a**) A simple contraction from left to right; (**b**–**e**) our two heuristics and the two other heuristics implemented in cotengra, specifically: (**b**) lookahead heuristic; (**c**) counting heuristic; (**d**) cotengra’s Betweenness heuristic; (**e**) cotengra’s RandomGreedy heuristic; (**f**) the heuristic used in [25], which exploits the fact that the tensor network originates from two circuits and contracts corresponding gates proportionally from the inside out.

To gain better insights into how these different heuristics work, we visualize the contraction order for the DJ circuit in Figure 9. The lookahead heuristic indeed spreads the contractions over the whole network more or less uniformly. As before, the counting heuristic imitates this behavior, although in a different order. The *Betweenness* heuristic starts on the very top of the network and sweeps to the bottom, while the *RandomGreedy* heuristic uses yet another order.

**Figure 10:**
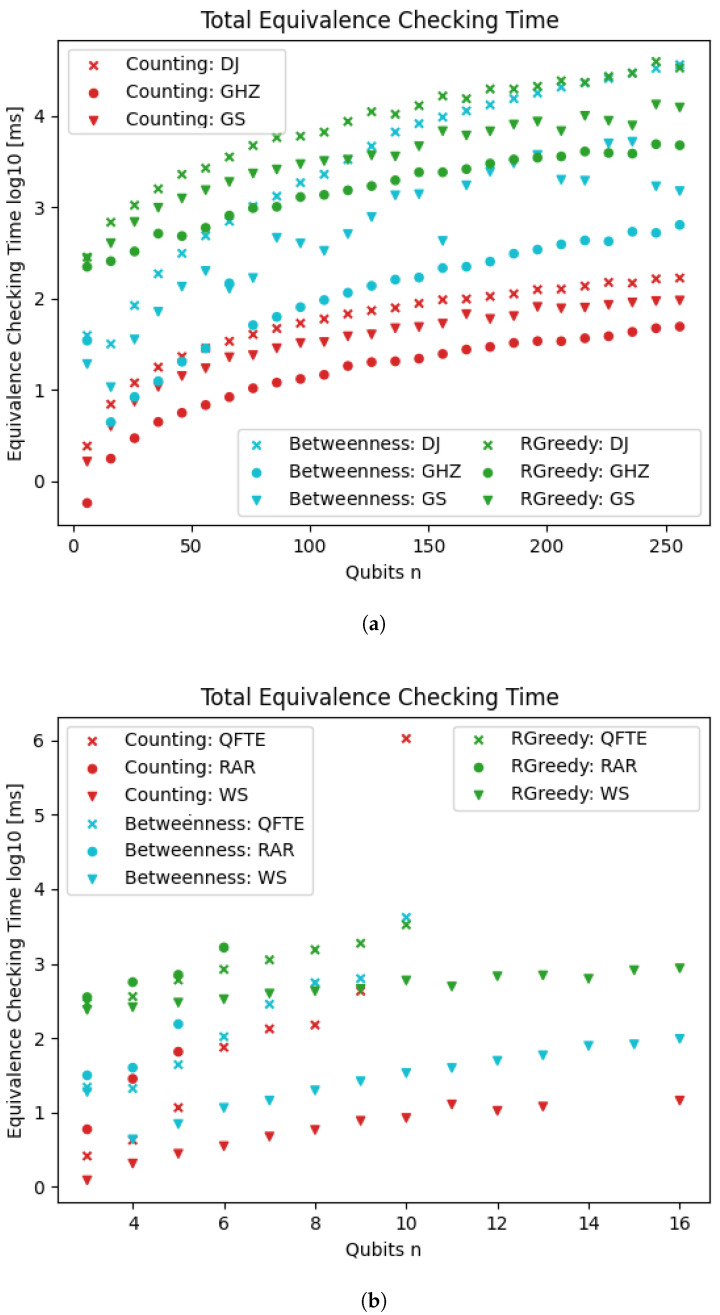
Equivalence checking time using our TDD-based approach with the counting heuristic and the heuristics implemented in cotengra. The times (y-axis; log-scale) are shown for six different families of quantum circuits (different markers) and for varying numbers of qubits (x-axis). (**a**) Three families of quantum circuits: DJ, GHZ, GS; (**b**) Three families of quantum circuits: QFTE, RAR, WS.

Figure 10 shows the results of the three evaluated heuristics for our application: the equivalence checking of quantum circuits. The results of the counting heuristic are identical to those presented in Figure 8. Of the two cotengra heuristics, Betweenness generally performs better than RandomGreedy.

Our counting heuristic consistently outperforms the cotengra heuristics on all three circuits by 1–2 orders of magnitude. This is surprising, as the cotengra heuristics are much more sophisticated. We conjecture that the advantage of the counting heuristic stems from two sources: the very efficient planning time and the specific application of quantum circuit equivalence. Studying the performance of our heuristics on further tensor network contraction benchmarks beyond our application is outside the scope of this paper.

### 4.4. Evaluation: Equivalence Checking of Quantum Circuits

As another baseline for our equivalence checking approach, we use qcec (Quantum Circuit Equivalence Checking) [24]. qcec is a C++ library and implements equivalence checking based on quantum decision diagrams (QDDs), which is similar in nature to our approach and thus makes for a good comparison [23]. qcec also uses ZX-calculus [27] in a preprocessing step, as it can sometimes very quickly prove circuit equivalence. Due to its fundamental difference and orthogonal performance, we deactivate the usage of ZX-calculus in the evaluation for a fair comparison to the QDD approach.

As outlined in Section 2.4, to check for the equivalence of two circuits $C_{1}$ and $C_{2}$, we ask whether the combined circuit $C_{1}\;C_{2}^{†}$ is the identity. We then transform the combined circuit into a tensor network and represent the tensors as TDDs, which we then contract using a contraction heuristic. Finally, we compare the result to the TDD for the identity matrix.

qcec’s strategy is related but differs in several aspects. First, it operates at the gate level and represents gates as QDDs. Second, it instead considers the circuit $C_{1}\;I\;C_{2}^{†}$ with an additional identity gate in the middle; then, it explores the circuit from the inside out, alternately multiplying one gate to the left or right in each step (see Figure 9f). Similar to our approach, the resulting QDD is finally compared to the QDD for the identity matrix.

**Figure 11:**
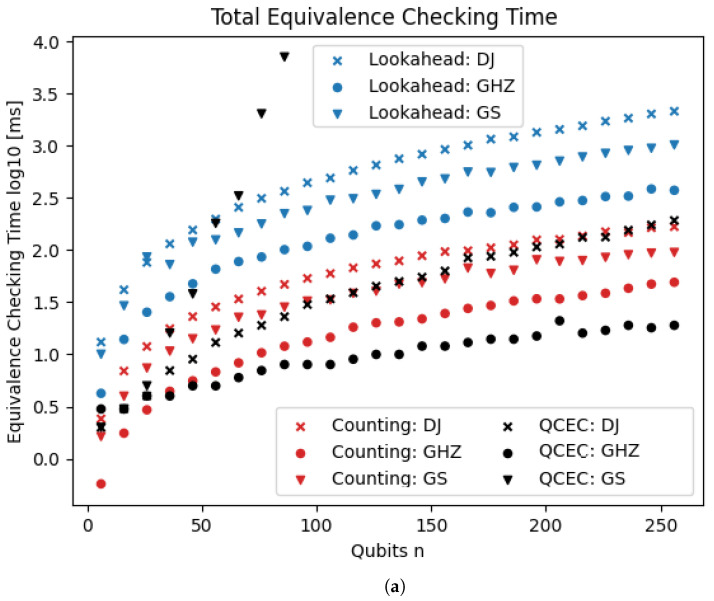

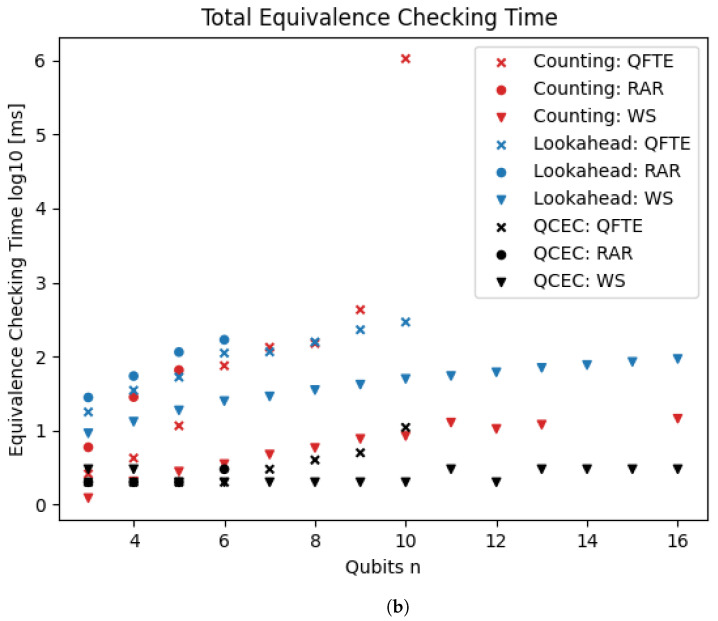
Equivalence checking time using our TDD-based approach with the counting heuristic and the QDD-based approach implemented in qcec. The times (y-axis; log-scale) are shown for six different families of quantum circuits (different markers) and for varying numbers of qubits (x-axis). (**a**) Three families of quantum circuits: DJ, GHZ, GS; (**b**) three families of quantum circuits: QFTE, RAR, WS.

Figure 11 shows the results of the comparison of our approach with qcec. For the first three quantum circuits (first plot), there is no clearly best approach, but the results are mostly consistent within the same family of circuits. qcec performs better on the GHZ circuit. For the DJ circuit, qcec is faster for small instances, but the run time grows faster than for the counting heuristic, which consequently outperforms on the largest instances. For the GS circuit, qcec only manages to get up to 96 qubits within a time limit of 1000 s, while our approach scale much better, easily reaching 256 qubits. On the second plot (some data points in Figure 11b (QFTE beyond 10 qubits; RAR beyond 6 qubits; WS for 14 or 15 qubits; and the counting heuristic) are missing due to issues with floating-point precision; this exemplifies that quantum computations are nontrivial to implement on a classical computer), qcec outperforms our approach on all circuits. Notably, the lookahead heuristic performs better than the counting heuristic on the QFTE circuit.

## 5. Conclusions

In this paper, we have described a framework for checking the equivalence of quantum circuits. In our framework, we reduce this problem to the contraction of a tensor network. We represent the tensor network efficiently using tensor decision diagrams (TDDs). As our main contribution, we have described the first contraction heuristics specifically designed for TDD networks. Our first heuristic, called the lookahead method, evaluates all possible next contractions with their effect on the TDDs and, in the end, picks the best contraction greedily. While the lookahead method performs very well, it also comes with a computationally heavy overhead. Our second heuristic, called the counting method, is designed to imitate the lookahead method while avoiding its overhead by distributing the contractions over the entire network.

In our experimental evaluation, we have demonstrated that our contraction heuristics outperform those implemented in the state-of-the-art library cotengra on this application. Moreover, we have demonstrated that equivalence checking via TDD networks is a viable approach that can sometimes outperform the QDD-based approach qcec.

There are several directions for future work. First, it would be interesting to study our contraction heuristics on other contraction benchmarks beyond quantum circuit equivalence, which we left open. Second, by studying the benchmarks where qcec performs better than our TDD-based approach, we hope to identify more powerful contraction heuristics. Third, our current implementation is purely sequential and does not exploit parallelization; modern hardware supports massive concurrent operations, and since our contraction heuristics operate in different regions of the TDD network, the computations would be easily parallelizable. Finally, we see great potential in finding contraction plans with the help of modern machine learning.

## Data Availability

Our implementation is openly available at https://github.com/Simonbolsen/P10 and https://github.com/ChBLA/TDDLinux (accessed on 1 November 2024).
